# NaPPOCON 2025: translating evidence into meaningful clinical practice in paediatric psycho-oncology—reflections from the First National Paediatric Psycho-Oncology Conference, 1–2 August 2025, Bangalore, India

**DOI:** 10.3332/ecancer.2026.2068

**Published:** 2026-01-30

**Authors:** Lakshmi R Varma, Krishna Gayathri Alakkal Rajesh, Sunil Bhat, Rhea Daruvala

**Affiliations:** Mazumdar Shaw Cancer Centre, Narayana Health, Bangalore 560099, India

**Keywords:** paediatric psycho-oncology, multidisciplinary care, psychosocial oncology

## Abstract

The National Paediatric Psycho Oncology Conference 2025 was the first such conference to be conducted in India with an exclusive focus on paediatric psycho-oncology. The conference brought together a multidisciplinary faculty coupled with participants from various specialities, including paediatric oncologists, surgical oncologists, radiation oncologists, nutritionists, nurses, psychologists, psychiatrists, social workers and trained psycho oncologists. This 2-day programme began with a hands-on skill-building workshop, followed by a day full of evidence-based, clinically applicable and research-rooted topics and discussions specific to various aspects of paediatric psycho-oncology. The added objective of focusing on applicability in low- and middle-income settings, while keeping cultural relevance in mindaided in making the discussions specific to the Indian context.

## Introduction

The National Paediatric Psycho Oncology Conference (NaPPOCON) 2025 was conducted on 1st and 2nd August at Narayana Health, Bangalore. This was the first national conference held nationally dedicated exclusively to paediatric psycho-oncology. The conference underscored the importance and recognition of psychosocial care in paediatric oncology as a vital component in providing holistic care for children with cancer and their families. The conference included professionals from various specialties who brought together multidisciplinary perspectives to further highlight the need for collaboration in order to provide optimal care. The professionals included paediatric oncologists, surgical oncologists, radiation oncologists, nutritionists, nurses, psychologists, psychiatrists, social workers and trained psycho-oncologists. The primary objective of the conference was to build skills and capacity in paediatric psycho oncology, sensitise professionals about the role of psychosocial care in paediatric oncology and amplify the perspectives from low- and middle-income countries such as India to aid in informing global paediatric psycho oncology discourse. The conference was endorsed, recognised and supported by various international and national bodies, including The International Society for Paediatric Oncology, The Indian Childhood Cancer Initiative, The Psycho Oncology Society of India and The Karnataka Paediatric Haematology Oncology Chapter.

## Day 1: Pre-conference workshop titled ‘Evidence to empathy: clinical skills for addressing procedural and treatment challenges in paediatric psycho-oncology’

On August 1st, 2025, the pre-conference workshop combined evidence-based learning with hands-on skill development. Sessions addressed common challenges in paediatric oncology settings such as procedural anxiety, pill swallowing, radiotherapy-related distress, feeding aversion and pain. Participants explored the psychological and physiological underpinnings of treatment-related distress and practised trauma-informed, developmentally sensitive interventions through case-based discussions and role plays. Training emphasised assessment tools, empathic communication and supportive strategies, including play therapy, distraction and parent coaching techniques. Case discussions and deliberations on ethical issues—such as treatment refusal, adolescent consent and cultural humility—further enriched the programme. The workshop concluded with a call for trauma-informed, multidisciplinary and child-centred care that combines clinical expertise with empathy while keeping a patient’s preferences in mind.

## Day 2: Proceedings from main conference on 2nd August 2025

**Figure 1.**
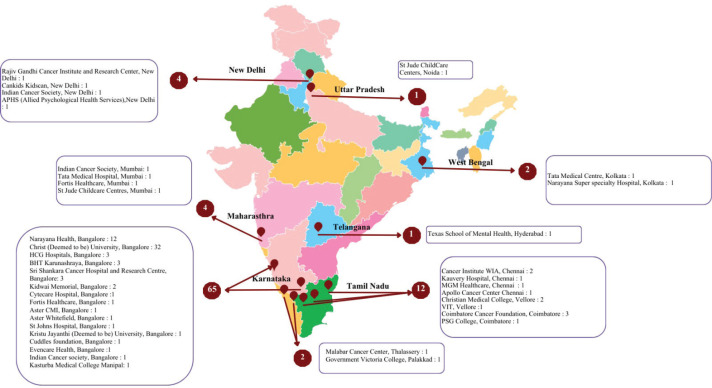
The geographical dispersion of participants from various Indian states.

The main conference day of NAPPOCON 2025 was met with enthusiasm and participants hailing from various multidisciplinary specialties. The conference received a total of 91 registrations from professionals across the country and from various professional backgrounds, including paediatric oncologists, surgical oncologists, radiation oncologists, nutritionists, nurses, psychologists, psychiatrists, social workers, psycho oncologists and trainees. The participants consisted of 31 professionals who were already working full time in the oncology space and 60 trainees. A brief overview of the distribution as per location is depicted in Figure 1 below.

### Session 1: the inaugural session: global perspectives and current initiatives in paediatric

Psycho-Oncology laid the foundation for the day by highlighting the evolution of psychosocial care worldwide over the years and current efforts in countries including India. The session brought together global insights and local initiatives aimed at integrating psychosocial support as a core element of paediatric oncology.

#### Session 1a

Look how far we’ve come! Evolution, development and integration of paediatric psycho-oncology into mainstream global care perspectives (*Speaker: Dr. Lori Wiener).*

In this pre-recorded talk, the speaker traced the evolution of paediatric psycho-oncology from the early recognition of psychosocial needs to its integration into multidisciplinary care. She highlighted global standards, supportive tools and collaborations that have advanced the field, while underscoring the need for equity, sustainability and culturally sensitive practices.

#### Session 1b

Current initiatives in promoting and standardising psycho-social care in paediatric oncology: The Indian scenario (*Speaker: Rhea Daruvala)*

Psychosocial care remains uneven across paediatric oncology centres in India. This talk highlighted national initiatives and collaborative efforts to develop evidence-based, culturally sensitive guidelines and establish standards of care while addressing challenges and opportunities for integrating psychosocial support into routine practice.

### Session 2: understanding the sychopathology, organicity and referrals

This session examined common psychological challenges in paediatric psycho-oncology, the differentiation between organic and functional disturbances, and the importance of timely specialist referrals. Three talks from multidisciplinary professionals highlighted their complementary roles in supporting children with cancer and their families in providing holistic care.

#### Session 2a

Common Psychological Challenges in PPO (*Speaker: Neha Tojan*)

The speaker highlighted the wide range of psychological challenges faced by children with cancer and their families, with studies showing high rates of depression, anxiety and post-traumatic stress. Concerns vary across ages and treatment phases, from tantrums and clinginess in younger children to identity struggles, social isolation and body image issues in adolescents. The talk stressed that psychosocial distress is inherent to the cancer experience, underscoring the need for timely, age-appropriate interventions.

#### Session 2b

Understanding psychopathology: Differentiating organicity from functional disturbances in children with cancer (*Speaker: Dr. Soumitra Datta)*

The talk addressed the challenge of distinguishing organic causes, such as treatment-related neurotoxicity, from functional or psychological symptoms, a frequent yet underexplored dilemma in practice. Case studies of seizure-like activity, pain amplification, paralysis and delirium highlighted the risks of misattribution, while speakers stressed the need for multidisciplinary collaboration, accurate screening and psychologically informed assessment. Dr. Datta also highlighted the role of a psychiatrist in the management of these symptoms.

#### Session 2c

A PHO’s perspective on paediatric psycho-oncology concerns: When do we make referrals to a specialist? (*Speakers: Dr. Suma)*

The session concluded with a paediatric oncologist’s perspective on the importance of psycho-oncology referrals. Key decision points across diagnosis, treatment, survivorship and end-of-life were outlined, highlighting the emotional needs of families and the role of specialist support at each stage of the cancer trajectory.

The session ended with an engaging Q&A, where the participants actively interacted with the faculty. Questions centred around differentiating treatment-related neurocognitive effects from psychological distress, deciding when to involve psychiatrists versus psychologists and addressing family resistance to referrals were some areas explored.

### Session 3: Debate — is reliance on clinical interview good enough or do you need more in paediatric psychosocial assessments?

The debate addressed a central methodological question in paediatric psycho-oncology: should psychosocial assessment rely solely on clinical interviews or should it routinely include standardised tools? Moderated by Dr. Brindha, the session featured two contrasting perspectives, giving the session a unique learning method for participants.

The first speaker, Pooja Chaudhry advocated for standardised assessments, emphasising their reliability, reduction of observer bias and value in longitudinal analysis, triaging and strengthening multidisciplinary care—particularly in high-volume oncology settings.

In contrast, Dr. Pavithra Harinath highlighted the irreplaceable role of clinical interviews, especially for children, where rapport, narrative exploration and contextual sensitivity are essential. She cautioned that standardised tools may miss culture-specific expressions of distress and oversimplify complex experiences.

The discussion concluded that both approaches are complementary. An integrated model, using structured tools for baseline screening and monitoring, alongside clinical interviews for depth and context, was seen as the most balanced and effective strategy.

### Session 4: interventions in paediatric psycho-oncology

This session focused on psychosocial care tailored according to the developmental stage of the patient and acknowledged that the intervention goals may vary significantly from toddler to young adults. Each speaker covered different stages, highlighting the key points to keep in mind for clinical practice.

#### Session 4a

Age is not just a number: Specialised interventions for toddlers and children *(Speaker Krishna Gayathri A R)*

This session focused on specialised interventions for toddlers and young children who were paediatricaborated on the significant psychosocial impact of cancer treatment and long hospitalisation in toddlers who have a limited cognitive and emotional capacity. Early planned interventions such as age-appropriate information, medical play, caregiver-aided psychoeducation and personalised procedural preparation can help lower the levels of acute procedural distress, prevent long-term anxiety and enhance adaptive coping.

#### Session 4b

Special considerations for intervention planning in AYAs *(Speaker: Aayushi Khaneja)*

The next talk in the session focused on supporting adolescents and young adults with developmentally-tailored psychosocial care and highlighted the complex interaction between the disease and developmental milestones, autonomy, identity and the importance of peer groups in this population. The talk also brought into perspective the prevalence of distress, anxiety, depression and social isolation in this population, and the importance of interventions that protect confidentiality, improve joint decision-making and improve skills in addressing sensitive topics such as fertility, body image and existential concerns.

#### Session 4c

Multidisciplinary care in paediatric oncology – Case-Based discussion *(Dr Saurabh, Dr. Pooja Mallya, Divya S, Vijay C, Sripriya Mahesh, Dr. Pavana)*

To conclude this session and provide practical examples of tailored care that is developmentally appropriate, a panel discussion with two cases was conducted. This panel also highlights how providing holistic care for children with cancer calls for a collaborative effort from various professionals to manage the intricate interconnections of the medical, psychological, developmental and social challenges faced by children. Collaborative decision making with families, sensitive care plans and coping capacity quality of life choices are enhanced by MDTs. Collaborative models aid in improving treatment adherence, reducing distress and instilling a sense of resilience in both patients and caregivers, highlighting their importance in paediatric oncology.

The cases consisted of a child with leukaemia receiving cranial radiation and an adolescent with osteosarcoma at risk of amputation. These two contrasting cases in terms of disease and age helped in highlighting how perspectives and approaches must change as professionals treat children with cancer. The discussion further emphasised that to navigate complicated cancer care journeys, a coordinated, well-informed team is essential. The discussion served as a practical framework for implementing MDT structures and workflow in Indian paediatric oncology services.

### Session 5: caring for caregivers

This session focused on two groups of caregivers in paediatric oncology: the family members and siblings who manage the emotional weight of the child’s illness along with social and psychological challenges, and the professionals who often work in high stress, emotionally overwhelming and high-morbidity environments.

#### Session 5a

Caring for the silent patient: Parents, caregivers and siblings *(Speaker: Aalapti Singh)*

This talk stressed that not all those affected by childhood cancer are in the spotlight. Parents, siblings and caregivers are often ‘collateral sufferers’ and absorb daily trauma while continuing to take on their families’ emotional, psychological, medical and financial needs together. They often experience grief, guilt and helplessness that are unrecognised. She emphasised the need for safe forums, proactive psychosocial screening that goes beyond just the patient and family-focused interventions that recognise their distinct but connected concerns as essential for holistic care.

#### Session 5b

Addressing stress and burnout in paediatric oncology professionals *(Speaker: Dr. Surendran Veeraiah)*

The important talk fostered a sense of shared experience among delegates and provided guidance for early career professionals, allowing them to reflect on their own skills of resilience and positive coping while working in paediatric oncology settings. Continuous engagement in this field requires the establishment of safe spaces for intentional self-care, structured peer support groups and continued supervision and mentorship. Professionals who work in the field are often significantly affected; hence, the need to continually address caregiver burnout becomes crucial in enhancing patient care and ensuring the reduction of professional burnout and attrition.

### Session 6: end of the cancer trajectory: survivorship or EOL

This session explored two critical but often less addressed stages in paediatric oncology, the transition from active treatment into survivorship and the need for high-quality end-of-life care, both highlighting the need for intentional planning, cultural alignment and multidisciplinary care at the end of the cancer trajectory.

#### Session 6a

Navigating the transition from treatment to survivorship: psychological support strategies* (Speaker: Revathy Rajagopal)*

This talk focused on transitioning into survivorship and provided insights into the various barriers that arise when children complete active treatment. Managing fears of recurrence and re-establishing school and social networks while managing physical effects are some major aspects of psychosocial adjustments involved in moving forward from treatment to the relative independence of survivorship. Psycho-oncologists play a crucial role in aiding the establishment of individual identity as well as a sense of communal belonging and adherence to maintenance and regular follow-ups.

#### Session 6b

Grief and bereavement in paediatric cancer: building legacies for children and their families at EOL *(Speaker: Michelle Normen)*

In the session, the speaker focused on the unique emotional, ethical and cultural components of providing care for children nearing the end of life. The importance of early palliative integration, open and age-appropriate communication and anticipatory guidance for both children and families were discussed. Aligning medical decisions with cultural and family-specific values and needs can significantly enhance dignity, minimise suffering and foster meaningful closure.

Both these talks reiterated the importance and need for paediatric psycho-oncology services at both ends of the cancer care spectrum, empowering life after cancer and acceptance for children and families.

### Session 7: specialised care for specialised situations

This session focused on the specialised psychosocial and functional challenges that arise in unique clinical situations. Experts shared their perspectives on addressing the needs of children undergoing bone marrow transplantation and onco-surgery.

#### Session 7a

Addressing BMT-specific psychosocial challenges in children undergoing transplantation* (Speaker: Lakshmi R. Varma)*

In the first talk of the session, the speaker elaborated on the various stressors present along the transplant trajectory that included pre-transplant anxiety, the emotional impact of long stay and isolation, the disruption of normal routines and challenges to family attachment styles as caregiving roles evolved. Tailored interventions utilised for each phase, such as one-on-one pre-bmt counselling sessions, parent support groups, neurocognitive assessments and fostering a sense of normalcy during transplantation were also discussed. Post-transplant, the focus on aspects such as reintegration in normal school and social life, regular follow-ups and adherence to precautions were highlighted.

#### Session 7b

Addressing functional difficulties of children undergoing surgery: special considerations for functional difficulties caused by onco surgery in children from a surgical oncologists perspective *(Speaker: Dr. Suman Byregowda)* and a psycho oncologists perspective *(Speaker: Dr. Brindha Sitaram)*

The session was divided into two perspectives. The first was a talk by a surgical oncologist who highlighted the challenges that manifest as part of onco-surgery in paediatric oncology. He elaborated on the complexities of surgical decision-making, especially in terms of the need to balance oncological clearance of disease versus functional preservation, quality of life and function. He further elaborated on the need for a multidisciplinary team during both the preparatory and postoperative phases of surgery. The importance of a close connection between multidisciplinary teams provides a nurturing space for anticipatory concerns, acceptance, rehabilitation and reintegration.

The second perspective of a psycho-oncologist presented the perspective on addressing challenges in onco-surgery. Some common concerns that arise are pre-operative anxiety, procedural distress, body image concerns and functional disabilities. Psychological inputs throughout the course of treatment can enhance and reinforce positive coping strategies and enhance communication among the families and the treating team. Integration of a multidisciplinary service aids in the child’s understanding and comfort with the treatment plan and in turn the outcomes.

The conference ended with an interactive quiz for participants to check their learning and understanding during the day, followed by feedback on the sessions and arrangements. The feedback received from the participants for the workshop is elaborated in Table 1, while the feedback for the main conference is further elaborated in Table 2 below.

## Conclusion

NaPPOCON 2025 successfully brought together a multidisciplinary community to advance psychosocial care as a core element in paediatric cancer treatment and established a much-needed national platform. The conference laid the foundation for shared professional identity within the field by combining scientific collaboration with skill-based workshops, case discussions and multidisciplinary dialogue. The future editions of NaPPOCON will focus on building specialised skills, expanding training avenues and facilitating knowledge exchange that is both evidence-driven and contextually relevant while continuing to advocate for standardised psychosocial care for children with cancer and their families.

## Conflicts of interest

The authors declare no conflicts.

## Funding

No funding declarations.

## Figures and Tables

**Table 1.** Feedback from participants for the workshop conducted on day 1.

Question	Response	Frequency
Overall quality	Excellent	24
	Good	13
Relevance to your professional	Extremely relevant	34
	Somewhat relevant	3
Expertise of speakers	Excellent	34
	Good	3
Depth of topics covered	Yes	33
	Somewhat	4
Balance between the theoretical and practical applications	yes	24
	Somewhat	13
Case discussion and interactive elements’ usefulness	Very useful	28
	Somewhat Useful	9
Effectiveness of workshop material	Very effective	25
	Adequate	12
Organisation and time management	Good	18
	Excellent	14
	Fair	5
Venue/seating/audio	Excellent	27
	Good	9
Workshop expectations were met?	Fully	34
	Partially	3

**Table 2.** The feedback from the participants on day 2 of the main conference.

Question	Response	Frequency
Overall quality of the conference	Excellent	39
	Good	8
	Fair	1
Relevance of session to professional interest	Extremely relevant	42
	Somewhat relevant	4
	Neutral	1
	-	1
Satisfaction about the range of topics covered	Very Satisfied	33
	Satisfied	12
	-	2
	Neutral	1
Formats most effective	Keynote and Lectures	37
	Panel	44
	Debate	27
	Q &A	11
Quality of speakers and presenters	Excellent	41
	Good	7
Opportunities for networking	yes	33
	Somewhat	15
Organisation and easy to follow	Yes	43
	Somewhat	5
Venue, seating, and technical arrangements	Excellent	33
	Good	14
	Fair	1
Registration and helpdesk	Smooth and efficient	47
	minor issues	1
Adequate information and communication	Yes	45
	Somewhat	3
